# Incorporating uncertainty measurement in the International Diabetes Federation Diabetes Atlas methodology for estimating global and national prevalence of diabetes in adults

**DOI:** 10.1186/2049-3258-73-S1-P31

**Published:** 2015-09-17

**Authors:** Katherine Ogurtsova, Leonor Guariguata, David Whiting, Nigel Unwin, Clara Weil, Joao Da Rocha Fernandes, Ute Linnenkamp, Nam H Cho, David Cavan, Lydia Makaroff

**Affiliations:** 1 International Diabetes Foundation, Brussels, Belgium; 2 Department of Preventive Medicine, Ajou University School of Medicine, Suwon, South Korea

## 

Diabetes is a major contributor to the global burden of mortality and morbidity. The International Diabetes Federation (IDF) uses a transparent reproducible methodology to generate global and country level estimates of diabetes prevalence in adults (20-79 years). However, the methodology produces point estimates and no uncertainty measurements have been reported.

To estimate the potential sources of uncertainty in the prevalence estimates and their magnitude, we performed two separate analyses: (1) a simulation study to access raw data uncertainty; (2) a bootstrap analysis of the sensitivity of the global prevalence estimate to the study selection process

In the simulation study, we drew 500 random samples inside of the 95% confidence interval (CI) range for each raw point estimate given in the data sources. Then, these samples were used in the IDF estimation procedure as conducted for the original data.

To access the sensitivity of a global probability magnitude to a selection of studies, we employed the bootstrap analysis. In a loop, one of all the selected studies was randomly picked up and removed from the list; the global prevalence was calculated on a base of remaining studies.

The simulation study produced an uncertainty interval 0.26% wide with the median shifted to the right due to low prevalence values. In the bootstrap analysis, the mean deviance from the point estimate was 0.006% (S.D. = 0.055%, 95% CI was 0.9% wide). However, the most important information was the extreme points in the analysis that composed 0.73% wide range. The total uncertainty interval was constructed as the united area of two measurements and was around 0.9% prevalence wide on the global level.

The uncertainty measurement will permit the comparison of IDF results with other sources and over time.

